# Predictive value of sperm DFI, ROS, and MMP for *In Vitro* fertilization pregnancy outcomes in asthenozoospermic patients

**DOI:** 10.3389/fendo.2026.1803726

**Published:** 2026-04-22

**Authors:** Sanhua Wei, Kaili Wang, Hongya Yang, Tao Yang, Kai Gao, Xuhui Ma, Ruonan Li, Zhenhua Chang, Zhenhua Lu, Hai Zhang, Xiaohong Wang

**Affiliations:** 1 Department of Obstetrics and Gynecology, Reproductive Medicine Center, Tang Du Hospital, Air Force Military Medical University, Xi’an, China; 2 National Translational Science Center for Molecular Medicine and Department of Cell Biology, Air Force Military Medical University, Xi’an, Shaanxi, China

**Keywords:** asthenozoospermia, DNA fragmentation index, mitochondrial membrane potential, pregnancy outcomes, reactive oxygen species

## Abstract

**Objective:**

To investigate the levels of sperm DNA fragmentation index (DFI), reactive oxygen species (ROS), and mitochondrial membrane potential (MMP) in asthenozoospermic patients and assess their predictive value for *in vitro* fertilization (IVF) pregnancy outcomes.

**Methods:**

This retrospective cohort study included 320 men with asthenozoospermic patients and 100 with normal semen parameters, assessed between January 2023 and June 2024. Of these, 143 couples undergoing IVF-embryo transfer were analyzed for outcomes. Semen samples were evaluated for conventional parameters, DFI, ROS, and MMP. Patients were stratified by DFI and MMP thresholds, and a combined DFI/MMP group was analyzed. Fertilization rate, cleavage rate, high-quality embryo rate, blastocyst formation rate, clinical pregnancy rate, miscarriage rate, and live birth rates were recorded. Multivariate logistic regression and receiver operating characteristic (ROC) curve analyses were performed.

**Results:**

Asthenozoospermic patients showed significantly higher DFI and ROS, and lower MMP compared to controls, with these parameters correlating to asthenozoospermia severity. DFI and MMP were identified as independent predictors of clinical pregnancy rate via multivariate logistic regression. The high DFI group had significantly lower fertilization, pregnancy, and live birth rates than the low DFI group, while the high MMP group demonstrated superior outcomes across all metrics compared to the low MMP group. The low DFI/high MMP group achieved the best results, exhibiting significantly higher fertilization, pregnancy, and live birth rates than the high DFI/low MMP group. ROC curve analysis indicated that the combination of DFI and MMP provided greater predictive power for clinical pregnancy (AUC = 0.771) than DFI (AUC = 0.643) or MMP (AUC = 0.651) alone.

**Conclusion:**

Sperm DFI and MMP are confirmed as independent and clinically significant predictors of clinical pregnancy in asthenozoospermic patients undergoing IVF treatment. The combined assessment of DFI and MMP offers superior predictive utility for IVF pregnancy outcomes compared to the assessment of either marker alone. These findings underscore the critical role of these sperm quality parameters in assessing reproductive potential and suggest significant clinical utility for guiding patient management and optimizing treatment strategies in assisted reproductive technologies.

## 1 Introduction

Approximately 10%–20% of couples of childbearing age worldwide experience infertility (1). Male factors contribute to 30%–50% of these cases (2). Over the past two decades, a significant declining trend in average sperm concentration and count has been observed globally. This persistent decline underscores the urgent need to investigate its underlying causes and implement strategies to prevent further deterioration of male reproductive health (3). Asthenozoospermia (AZS), characterized by reduced sperm motility, is a prevalent finding and affects approximately 82% of infertile men (4).

AZS is characterized by reduced sperm motility—a progressive motility (PR) under 32% or total motility (PR+NR) below 40%. It is a common male infertile factor, often coexisting with abnormal sperm count or morphology, but its causes are frequently idiopathic (5). Contributing factors include cryptorchidism, varicocele, infections, hormonal disturbances, environmental toxins, chemotherapy, lifestyle, and genetics (6).

Sperm DFI measures the proportion of sperm with DNA breaks. It serves as a sensitive indicator of both DNA damage and sperm quality and is increasingly used in fertility assessment (7). Elevated DFI correlates with poorer ART outcomes (8–10). ROS, such as superoxide, hydroxyl radicals, and hydrogen peroxide, are by-products of sperm metabolism. While physiological ROS levels aid in capacitation and motility, excessive ROS cause DNA damage, membrane disruption, and mitochondrial impairment (11, 12). Mitochondria in the sperm midpiece are crucial for energy production, fueling hyperactivation, capacitation, acrosome reaction, calcium balance, and apoptosis (13, 14). MMP, the transmembrane electrical gradient across the inner mitochondrial membrane, reflects mitochondrial functional integrity and is a key indicator of sperm viability and fertilization potential (15, 16).

The PR of sperm following preparation has been identified as a critical factor for the success of intrauterine insemination (IUI). Specifically, sperm motility exceeding 40% is considered a favorable prognostic indicator for IUI (17). Similarly, the percentage of morphologically normal sperm significantly influences the fertilization capacity in IVF treatment. A reduced normal morphology rate of less than 4% is associated with a significantly diminished IVF fertilization rate compared with baseline levels (18). However, some research has indicated that conventional semen parameters, including sperm morphology, may not reliably predict ART outcomes in infertile males (19–21), highlighting the need for more sensitive indicators. Emerging evidence suggests that the sperm DFI may hold prognostic value for ART outcomes (8). Furthermore, compared with standard sperm parameters, the combined assessment of sperm DFI and MMP has shown promise for predicting natural conception rates more effectively (22). Despite these advancements, discrepancies persist across reported findings. The present study aims to investigate the clinical utility and predictive value of sperm DFI, ROS, and MMP assays in patients diagnosed with AZS, specifically in relation to IVF pregnancy outcomes.

## 2 Methods

### 2.1 Study population and inclusion/exclusion criteria

This study recruited 320 patients with AZS of varying severity (mild, moderate, severe) and included 100 healthy men as controls. All participants were excluded for reproductive system malformations, severe chronic diseases, endocrine abnormalities, urogenital infections, adverse environmental exposures, genetic disease history, or hormone therapy/chemoradiotherapy history.

The study retrospectively analyzed data from couples where the male partner had AZS and underwent *in vitro* fertilization-embryo transfer (IVF-ET). Data collected included age, body mass index (BMI), duration of infertility for both partners, semen parameters, and IVF-ET outcomes. Based on sperm DFI, ROS, and MMP levels, patients were divided into high and low groups for single or combined indicators.

**Figure 1:**
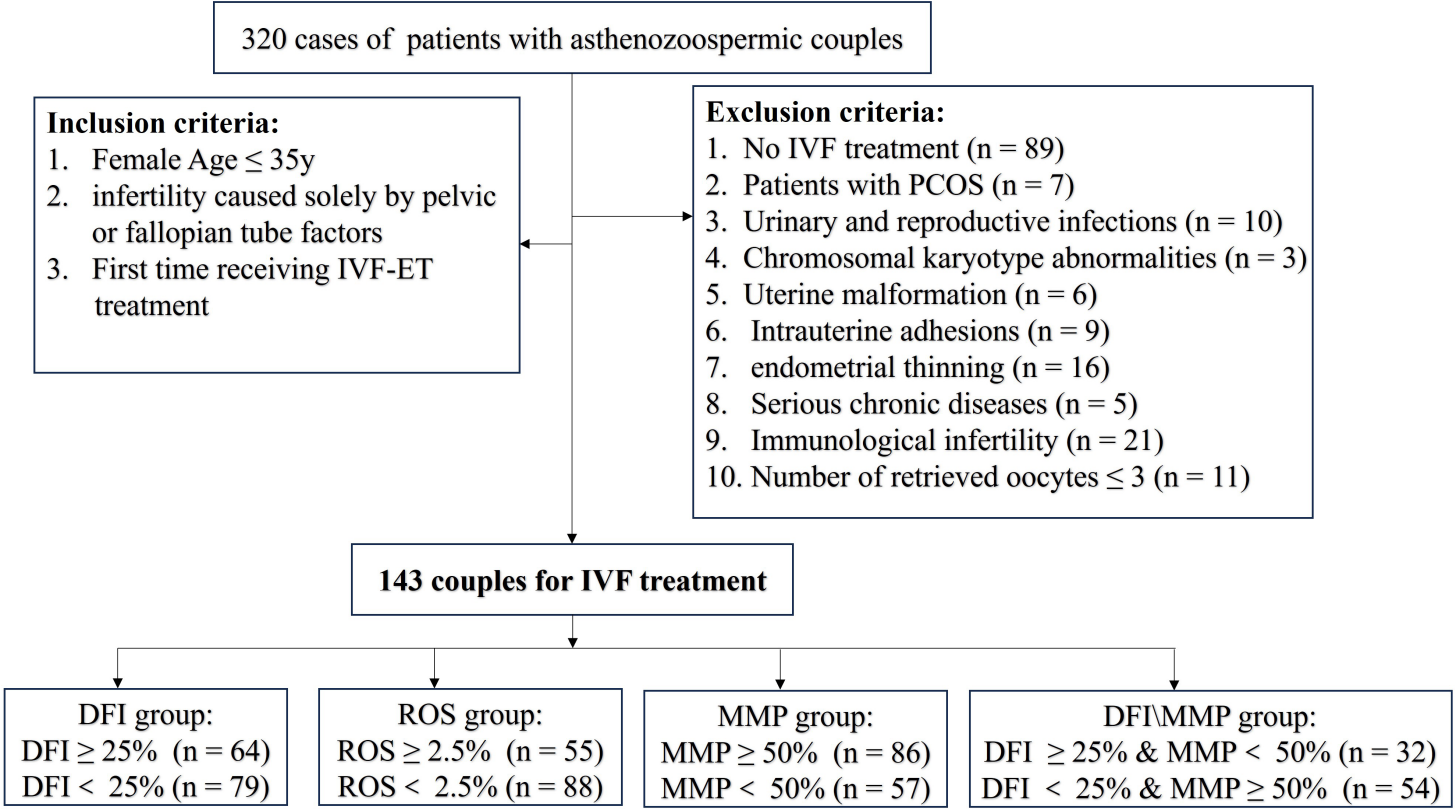
Flowchart of patient inclusion and exclusion. IVF, *in vitro* fertilization; PCOS, polycystic ovary syndrome; DFI, DNA fragmentation index; ROS, reactive oxygen species; MMP, Mitochondrial membrane potential.

To control for confounding factors, female partners were required to be ≤35 years old, have infertility solely due to pelvic or fallopian tube factors, and be undergoing their first IVF-ET cycle. Couples with chromosomal abnormalities in either partner, polycystic ovary syndrome (PCOS) in the female partner, uterine issues, infections, severe systemic diseases, immunological infertility, or a first IVF cycle with ≤3 retrieved oocytes were excluded. Ultimately, according to the screening process illustrated in **Figure 1**, 143 couples were included in the final analysis.

### 2.2 Semen collection and conventional semen analysis

After a sexual abstinence period of 2–7 days, semen samples were collected by masturbation. The freshly collected samples were placed on a warming plate for liquefaction prior to analysis. A routine semen analysis was performed, which involved computer-assisted semen analysis (CASA) for assessing sperm concentration, total sperm count, total motility, and PR. Sperm morphology was evaluated manually under a microscope using Papanicolaou-stained preparations, and the percentage of normal forms was calculated.

### 2.3 Sperm DFI, ROS, and MMP assays

Sperm parameters—including DFI, ROS levels, and MMP—were assessed using flow cytometry in conjunction with specific fluorescent staining kits (BoruiDe, Shenzhen). DNA fragmentation was evaluated via the Sperm Chromatin Structure Assay (SCSA). Intracellular ROS levels were quantified using the cell-permeable fluorescent probe 2′,7′-dichlorodihydrofluorescein diacetate (DCFH-DA). Mitochondrial membrane potential (MMP) was assessed using JC-1 staining. All analyses were performed on a CytoOne E6 flow cytometer (Mindray, Shenzhen). Following semen liquefaction, sperm concentration (×10^6^/mL) was determined for each sample. Aliquots of prepared sperm suspensions were then subjected to the respective staining protocols. For each assay, a minimum of 5,000 spermatozoa per sample were analyzed to determine the relevant parameters. All samples were analyzed in triplicate, and the mean values were used for final data interpretation.

### 2.4 Definition and calculation of analytical parameters

Semen parameters (age, abstinence duration, volume, concentration, PR, NP, total motility, DFI, ROS, MMP) were compared between normal and AZS groups. Differences in DFI, ROS, and MMP across AZS severity levels were assessed, along with their correlations with conventional semen parameters. Female characteristics and ART outcomes were compared among groups. Embryo development indicators included fertilization rate, cleavage rate, high-quality embryo rate, and blastocyst formation rate. Pregnancy outcomes comprised clinical pregnancy rate, miscarriage rate, and live birth rate.

### 2.5 Statistical analysis

Statistical analysis was performed using SPSS 26.0. Normally distributed data are presented as mean ± SD and compared using t-tests or ANOVA. Non-normally distributed data are expressed as median (IQR) and analyzed with non-parametric tests. Correlations were assessed using Spearman’s rank correlation. Multivariate logistic regression was used to analyze the association of DFI, ROS, and MMP with clinical pregnancy rates. ROC curve analysis evaluated the predictive value of DFI, MMP, and their combination for pregnancy outcomes, with the AUC indicating predictive efficacy. Optimal thresholds were determined by maximizing the Youden index. A p-value < 0.05 was considered statistically significant.

## 3 Results

### 3.1 Comparison of general characteristics and semen parameters between patients with AZS and healthy men

We compared the basic characteristics and routine semen parameters between 320 AZS patients and 100 healthy men (**Table 1**). The two groups were comparable in terms of BMI, abstinence duration, and semen volume. However, the AZS group was significantly older than the control group. Analysis of semen parameters revealed that the sperm concentration, total sperm count, total motility, PR, and percentage of sperm with normal morphology were significantly lower in the AZS group than in the healthy control group. Measurements of the sperm DFI, ROS levels, and MMP in patients with AZS and in healthy men revealed that the DFI and ROS levels in the latter group were significantly lower than those in the AZS group, whereas MMP was significantly higher. These findings indicate that the DFI, ROS, and MMP can serve as auxiliary indicators for evaluating AZS. Further correlation analysis performed among DFI, ROS, and MMP levels in AZS patients revealed that these three sperm parameters were all correlated with the severity of AZS (**Supplementary Figure** and **Supplementary Table 1**). This suggests that in patients with severe AZS, notable abnormalities in DFI, ROS, and MMP occur, which are likely associated with impaired sperm quality and function.

**Table 1:** Comparison of male characteristics and semen parameters between AZS patients and healthy controls.

Characters	Healthy men (n=100)	AZS (n=320)	Z/t	*p*
Age (years)	33.0 ± 5.0	34.3 ± 5.1	-2.280	0.023
BMI (kg/m²)	24.6 ± 2.3	24.9 ± 2.0	-1.072	0.285
Abstinence time (days)	4.2 ± 3.2	4.3 ± 1.7	-0.519	0.604
Semen volume (mL)	3.5 (2.6,5.6)	4.2 (2.9,5.6)	0.235	0.815
Semen concentration (10^6^/ml)	67.8 (45.5,110.9)	42.5 (19.0,83.3)	-3.862	< 0.001
Total sperm count (10^6^/per ejaculation)	251.3 (154.5,424.5)	151.3 (74.1,325.5)	-3.927	< 0.001
Progressive motility (%)	48.6 ± 10.6	16.3 ± 8.8	27.540	< 0.001
Sperm motility (%)	60.4 ± 11.7	23.5 ± 11.0	28.719	< 0.001
Normal form percentage (%)	7.0 (5.0, 8.0)	5.0 (4.0, 7.0)	-3.703	< 0.001
DFI (%)	10.3 (6.9, 16.5)	27.0 (17.4, 43.2)	9.091	< 0.001
ROS (%)	1.0 (0.6, 2.0)	2.3 (1.2, 4.4)	7.461	< 0.001
MMP (%)	68.9 (58.5, 76.4)	52.3 (39.3, 63.0)	-8.078	< 0.001

Data are expressed as mean (standard deviation), median (interquartile range), or number (percent), as appropriate. BMI, body mass index; DFI, DNA fragmentation index; ROS, reactive oxygen species; MMP, Mitochondrial membrane potential.

### 3.2 Correlation between the sperm DFI, ROS levels, and MMP and standard semen parameters in patients with AZS

We assessed the associations between DFI, ROS, MMP, and conventional semen parameters in AZS patients (**Table 2**). DFI and ROS levels were positively correlated with patient age. Conversely, DFI was negatively correlated with PR, total motility, and normal morphology. Higher ROS levels were also negatively correlated with sperm concentration, PR, total motility, and normal morphology. In contrast, higher MMP positively correlated with all these parameters. DFI, ROS, and MMP were significantly interrelated (**Supplementary Table 2**). DFI and ROS were positively correlated, while both were negatively correlated with MMP.

**Table 2:** Correlation analysis between sperm DFI, ROS, MMP levels and standard semen parameters in AZS patients.

Characters	DFI		ROS		MMP	
	*r_s_*	*p*	*r_s_*	*p*	*r_s_*	*p*
Age (years)	0.163	0.001	0.143	0.003	-0.026	0.592
BMI (kg/m²)	0.041	0.408	0.005	0.926	-0.015	0.760
Abstinence period (days)	0.080	0.101	-0.069	0.156	0.093	0.065
Semen volume (mL)	0.064	0.254	0.023	0.638	0.016	0.744
Semen concentration (10^6^/ml)	0.082	0.094	-0.483	< 0.001	0.563	< 0.001
Progressive motility (%)	-0.479	< 0.001	-0.488	< 0.001	0.523	< 0.001
Sperm motility (%)	-0.468	< 0.001	-0.506	< 0.001	0.554	< 0.001
Normal morphology rate (%)	-0.105	0.031	-0.371	< 0.001	0.393	< 0.001

*p* < 0.05 represents significant difference.

### 3.3 Multivariate logistic regression analysis of factors affecting the IVF clinical pregnancy rate

To evaluate the determinants of the IVF clinical pregnancy rate, a binary logistic regression analysis was employed that incorporated male age, the DFI, ROS, and MMP as independent variables. The analysis revealed that both DFI (OR = 1.108, 95% CI: 1.074–1.142, *p* = 0.000) and MMP (OR = 1.335, 95% CI: 1.058–1.686, *p =* 0.000) were independent influencing factors. Conversely, neither male age (OR = 0.993, 95% CI: 0.936–1.054, *p* = 0.827) nor ROS (OR = 0.953, 95% CI: 0.993–0.974, *p* = 0.055) demonstrated a statistically significant independent association with the outcome (**Table 3**).

**Table 3:** Multivariate logistic regression analysis identifying sperm DFI and MMP as independent predictors of clinical pregnancy after IVF.

Variable	β	OR	95%CI	*p*
Age	-0.007	0.993	(0.936, 1.054)	0.827
DFI	0.102	1.108	(1.074, 1.142)	0.000
ROS	0.289	0.953	(0.933, 0.974)	0.055
MMP	-0.048	1.335	(1.058, 1.686)	0.000

β, regression coefficient; OR, odds ratio; 95% CI, 95% confidence interval. *P* < 0.05 represents significant differences.

### 3.4 Correlation analysis between the sperm DFI, ROS, and MMP and embryonic development and IVF pregnancy outcomes

This study included 143 ART cycles, with participants stratified into groups based on the sperm DFI, ROS, and MMP: high DFI (≥ 25%, n = 64) vs. low DFI (< 25%, n = 79); high ROS (≥ 2.5%, n = 55) vs. low ROS (< 2.5%, n = 88); and high MMP (≥ 50%, n = 86) vs. low MMP (< 50%, n = 57). No significant differences in female baseline characteristics were found among these groups (**Supplementary Table 3**) except estradiol (E2). Compared with the low DFI group, the high DFI group had significantly lower fertilization (72.3% vs. 80.3%), pregnancy (45.3% vs. 63.2%), and live birth (34.4% vs. 57.0%) rates (*p* = 0.000, *p* = 0.032 and *p* = 0.004). However, the cleavage rate, high-quality embryo rate, blastocyst formation rate, and miscarriage rate did not significantly differ between the two groups. By contrast, no significant differences in any embryological or clinical outcome parameters were observed between the high- and low-ROS groups. Compared with the low-MMP group, the high-MMP group had significantly higher fertilization (78.2% vs. 74.1%), pregnancy (63.9% vs. 42.1%), and live birth (54.7% vs. 35.1%) rates (*p* = 0.044, *p* = 0.010 and *p* = 0.022). The cleavage rate, high-quality embryo rate, blastocyst formation rate, and miscarriage rate did not significantly differ (**Table 4**). Our analysis identified that sperm DFI and MMP are correlated with key IVF outcomes, including the fertilization rate, pregnancy rate, and live birth rate, whereas ROS levels within the studied range were not significantly associated.

**Table 4:** Comparison of embryonic development and IVF pregnancy outcomes between high DFI\ROS\MMP groups and low DFI\ROS\MMP groups.

Indicators	High DFI group	Low DFI group	χ^2^	*p*	High ROS group	Low ROS group	χ^2^	*P*	High MMP group	Low MMP group	χ^2^	*p*
	(≥ 25%)	(< 25%)			(≥ 2.5%)	(< 2.5%)			(≥ 50%)	(< 50%)		
cases	n=64	N=79			n=55	n=88			n=86	n=57		
Fertilization rate (%)	72.3	80.3	16.136	< 0.001	78.7	75.3	1.756	0.185	78.2	74.1	4.038	0.044
	(622/860)	(764/951)			(515/654)	(871/1157)			(839/1073)	(547/738)		
Cleavage rate (%)	97.9	96.5	2.551	0.110	97.5	96.9	0.383	0.536	97.7	96.2	2.929	0.087
	(609/622)	(737/764)			(502/515)	(844/871)			(820/839)	(526/547)		
High-quality embryo rate (%)	42.5	41.7	0.104	0.747	43	41.5	0.314	0.575	40.1	45.1	3.203	0.074
	(259/609)	(307/737)			(216/502)	(350/844)			(329/820)	(237/526)		
Blastocyst formation rate (%)	53.8	59	2.777	0.096	54.7	57.7	0.847	0.357	56.1	54.2	0.082	0.775
	(262/487)	(308/522)			(220/402)	(350/607)			(339/604)	(231/405)		
Pregnancy rate (%)	45.3	63.2	4.622	0.032	49	59	1.369	0.242	63.9	42.1	6.618	0.010
	(29/64)	(50/79)			(27/55)	(52/88)			(55/86)	(24/57)		
Miscarriage rate (%)	23.3	10	1.673	0.196	14.8	15.4	0.004	0.947	14.5	16.7	0.058	0.809
	(7/30)	(5/50)			(4/27)	(8/52)			(8/55)	(4/24)		
Live birth rate (%)	34.4	57	8.077	0.004	41.8	50	0.910	0.340	54.7	35.1	5.269	0.022
	(22/64)	(45/79)			(23/55)	(44/88)			(47/86)	(20/57)		

Data are expressed as number (percent), *P* < 0.05 represents significant differences.

### 3.5 ROC curve analysis of the sperm DFI and MMP in predicting IVF pregnancy outcomes

To evaluate the predictive power of the sperm DFI and MMP on IVF pregnancy outcomes, receiver operating characteristic (ROC) curve analysis was performed. Our findings indicated that both DFI and MMP are independent risk factors for clinical pregnancy rates. Further comparisons between the high-MMP/low-DFI group and the high-DFI/low-MMP group revealed that the low-DFI/high-MMP cohort experienced significantly superior fertilization, pregnancy, and live birth rates (**Supplementary Table 4**). Consequently, we investigated the value of the DFI, MMP, and their combined use in predicting IVF pregnancy success.

**Figure 2:**
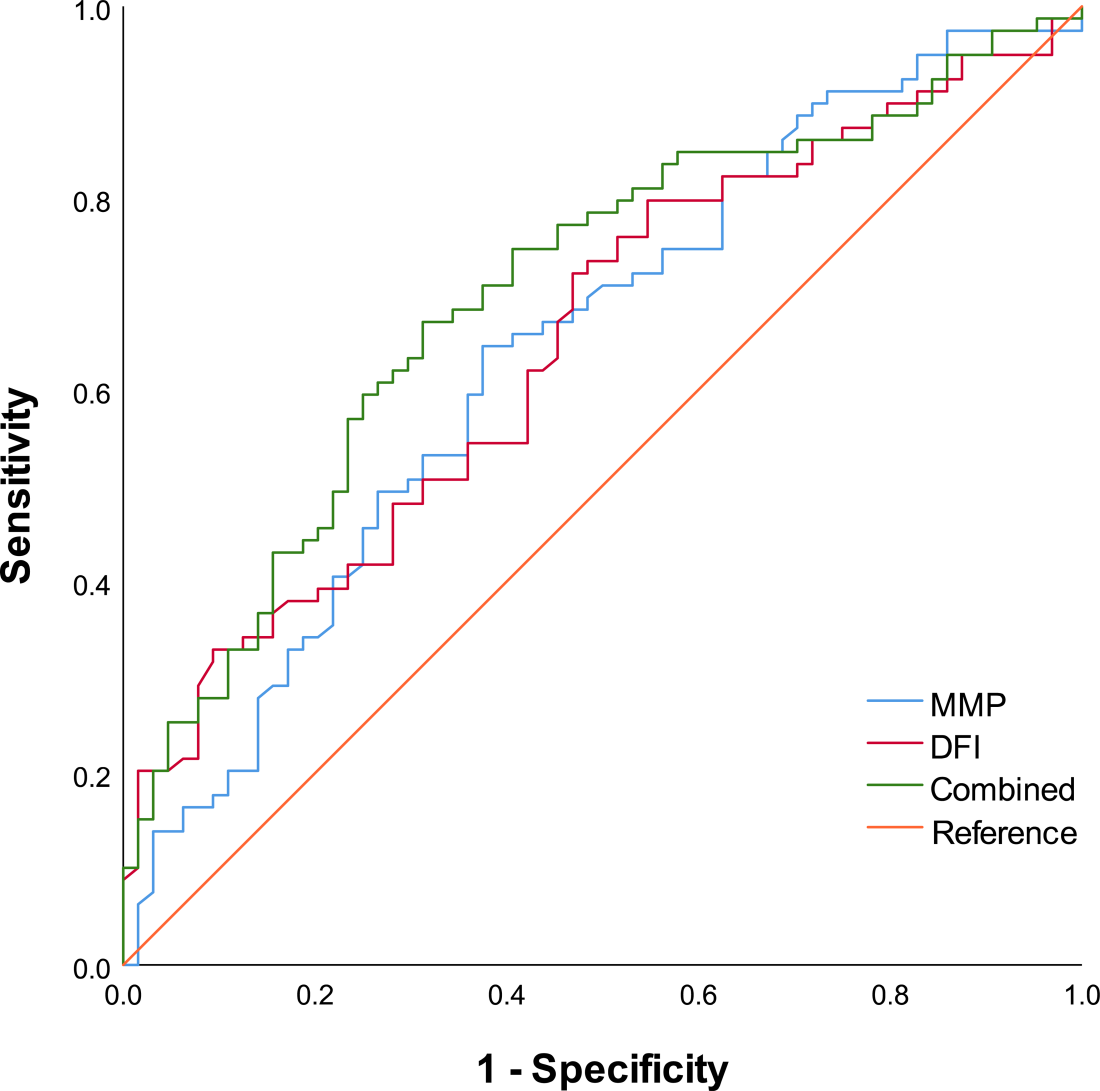
ROC curve analysis of sperm DFI and MMP for predicting clinical pregnancy in IVF cycles. The area under the curve (AUC) values with 95% confidence intervals are shown for DFI (red line), MMP (blue line), and their combination (green line).

The results of the ROC analysis revealed that the sperm DFI alone had a predictive AUC of 0.643 (*p* = 0.002, 95% CI: 0.560–0.739), with an optimal cut-off value of 0.284, yielding a sensitivity of 0.722 and a specificity of 0.531. Similarly, sperm MMP had a predictive AUC of 0.651 (*p* = 0.003, 95% CI: 0.551–0.733), with a cut-off value of 0.539, a sensitivity of 0.646, and a specificity of 0.625. Notably, when the DFI and MMP were used in combination for joint prediction, the AUC significantly improved to 0.771 (*p* = 0.000, 95% CI: 0.615–0.787), with a sensitivity of 0.671 and a specificity of 0.687. These findings indicate that compared with either DFI or MMP individually, the combined prediction model offers superior predictive accuracy (**Table 5**; **Figure 2**).

**Table 5:** Valuation the predictive power of the sperm DFI and MMP for IVF pregnancy outcomes.

Indicators	Cut-off	*AUC*	Youden’s index	Sensitivity		Specificity		95%CI	*p*
DFI	0.284	0.643	0.253		0.722	0.531	0.560-0.739		0.002
MMP	0.539	0.651	0.271		0.646	0.625	0.551-0.733		0.003
Joint diagnosis		0.771	0.358		0.816	0.785	0.615-0.787		< 0.001

AUC, Area Under the Curve; 95% CI, 95% confidence interval. *P* < 0.05 represents significant differences.

## 4 Discussion

The sperm DFI, ROS levels, and MMP are indicators of sperm genetic stability, oxidative stress status, and energy metabolism level, respectively. Measuring these parameters holds significant guiding value for a deeper understanding of sperm function and male reproductive potential. Our study findings indicate that compared with healthy men, patients with AZS have a lower sperm concentration, motility, percentage of PR, and normal sperm morphology. These findings suggest that AZS patients not only have impaired sperm motility but are also likely to present with abnormalities in sperm quantity and morphology, which is consistent with the common clinical observation of oligospermia or teratozoospermia in these individuals (6). Furthermore, these parameters were also correlated with the severity of AZS. Thus, in addition to conventional semen analysis and sperm morphology assessments, the DFI, ROS levels, and MMP can serve as valuable biomarkers to further assess semen quality.

A correlation analysis revealed that the sperm DFI and ROS levels were positively correlated with patient age (**Table 2**) but negatively correlated with sperm motility, PR, and normal morphology. Conversely, sperm MMP was positively correlated with the sperm concentration, motility, PR, and normal morphology. These findings align with the established consensus that ageing significantly impairs male sperm quality (23, 24), a phenomenon further underscored by the higher average age observed in the AZS group than in the healthy control group. Notably, prior research has demonstrated that the DFI of males older than 50 years is 4.58 times greater than that of males younger than 30 years (25), corroborating our observation of a strong positive correlation between age and sperm DNA damage.

DFI, ROS, and MMP are interrelated. We also conducted correlation analysis and found that DFI and ROS levels were positively correlated, while MMP was negatively correlated with DFI and ROS levels. Some research indicated that ROS directly damage mitochondria, leading to reduced MMP and apoptosis, which in turn exacerbates ROS production, forming a feedback loop detrimental to sperm quality and male fertility (26). Mechanistically, elevated miR-151a-5p in high-DFI samples has been shown to induce MMP depolarization, ATP depletion, a ROS surge, and apoptosis (27), supporting a “high DFI→ mitochondrial dysfunction→ ROS burst” pathway. Furthermore, MMP depletion precedes DFI elevation in boar semen preservation, suggesting that MMP is an early indicator of oxidative stress (28).

The precise influence of BMI on semen quality remains inconclusive (29, 30). While previous research involving sperm donors has linked both low and high BMI to reduced semen quality (31), our analysis revealed no significant difference in BMI between patients with normal sperm motility and those with AZS. Moreover, we observed no significant correlations between BMI and the DFI, ROS levels, or MMP. It is possible that BMI must reach a more extreme threshold to noticeably affect these specific parameters, and our study cohort may not have included a sufficient number of individuals with such BMI levels to detect an effect.

Spermatozoa are highly susceptible to DNA damage because of their lack of repair mechanisms, which can impair embryonic development and ART outcomes if the repair capacity of the oocyte is overwhelmed (32). In our cohort, the DFI was an independent risk factor for clinical pregnancy, with the high-DFI group exhibiting significantly lower fertilization, pregnancy, and live birth rates. No significant differences were found in other embryo development parameters or miscarriage rates, although the latter showed a considerable increase (23.3% vs. 10%), underscoring the potential clinical significance of larger-scale validation. These results support several studies (8, 33–35) but conflict with others (36–38), a controversy potentially explained by heterogeneity in study populations and methodologies. Despite this controversy, a high DFI is consistently associated with miscarriage risk in patients with recurrent pregnancy loss (39), and novel indicators such as DNA breakpoints (MDB) are being explored for prediction (40).

Elevated levels of ROS are detected in approximately 25%–40% of infertile men (41). While the addition of antioxidants to frozen sperm has been shown to improve motility and antioxidant capacity post-thaw, as well as increase cleavage and blastocyst formation rates (42), and oral antioxidant therapy conceptually improves semen quality and fertilization rates (43), a double-blind clinical trial demonstrated no improvement in IVF outcomes with vitamin E supplementation (44). Consistent with the latter findings, our research revealed no significant differences in female general characteristics between the high- and low-ROS groups (**Supplementary Table 3**), nor did we observe disparities in embryonic development or IVF pregnancy outcomes. Multivariate logistic regression analysis further supported this finding, indicating that ROS is not an independent risk factor for clinical pregnancy. Consequently, considerable uncertainty persists regarding the correlation between the levels of oxidative stress markers in sperm and IVF pregnancy outcomes.

The unique structure of mature sperm mitochondria plays a crucial role in sperm vitality, capacitation, and fertilization ability (45). While early studies indicated no significant correlation between MMP and ICSI outcomes (46), recent research widely suggests an association between MMP and fertilization rates, as well as ART outcomes (47, 48). Notably, high MMP has even been reported to increase fertilization rates in IVF treatment (49), and combined testing of the sperm DFI and MMP can improve the prediction of natural conception (22). Our findings are consistent with this latter trend, with no significant differences in general participant information between the high- and low-MMP groups. Crucially, compared with the low-MMP group, the high-MMP group had higher fertilization, pregnancy, and live birth rates, although the cleavage, high-quality embryo, blastocyst formation, and miscarriage rates remained comparable. To further support the significance of MMP, multivariate logistic regression analysis revealed it as an independent risk factor affecting clinical pregnancy rates.

To assess the impact of sperm DFI, ROS, and MMP on IVF outcomes, women under 35 with tubal/pelvic infertility were enrolled. Baseline female characteristics were consistent across groups (except E2, **Supplementary Table 3**), minimizing confounding. Intervals between semen analysis and hCG administration were controlled to ensure accurate semen parameter reflection. Results showed that elevated DFI was linked to lower fertilization, clinical pregnancy, and live birth rates, while high MMP correlated with improved outcomes. Sperm ROS did not significantly affect early embryo development or IVF pregnancy rates. This is likely because DFI and MMP directly reflect sperm functional integrity, whereas ROS are upstream factors that can damage sperm. High ROS can lead to increased DFI, reduced MMP, and impaired motility (50, 51). Thus, DFI and MMP may be more direct predictors of IVF success. The study measured live sperm with high ROS proportions, not absolute concentrations, and observed that ROS-tolerant sperm might exhibit better motility and resistance (41, 52), potentially explaining the lack of association of ROS with IVF outcomes.

We analyzed embryonic development and IVF pregnancy outcomes by comparing patients with low DFI/high MMP to those with high DFI/low MMP. Compared with the high DFI/low MMP group, the low DFI/high MMP group demonstrated significantly higher fertilization, pregnancy, and live birth rates, although the case numbers were modest (**Supplementary Table 4**). Further evaluation using ROC analysis revealed that sperm DFI alone predicted clinical pregnancy with an AUC of 0.643 (sensitivity 72.2%, specificity 53.1%), and that sperm MMP alone predicted clinical pregnancy with an AUC of 0.651 (sensitivity 64.6%, specificity 62.5%). The combined detection of sperm DFI and MMP significantly improved this predictive capability, achieving an AUC of 0.771 with a sensitivity of 81.6% and a specificity of 78.5%. These findings indicate that the synergistic assessment of sperm DFI and MMP is highly valuable for predicting IVF pregnancy outcomes in assisted reproductive treatments.

## 5 Conclusion

This study confirms that the sperm DFI and MMP correlate with conventional semen parameters in AZS patients and may have a predictive value for IVF pregnancy outcomes. Nevertheless, to fully establish their clinical utility for assessing male infertility and predicting IVF pregnancy outcomes, further rigorous prospective studies are needed for continued exploration and validation.

## Data Availability

The original contributions presented in the study are included in the article/**Supplementary Material**. Further inquiries can be directed to the corresponding author.
