# Supplementary material for: Predictive value of sperm DFI, ROS, and MMP for In Vitro fertilization pregnancy outcomes in asthenozoospermic patients
Source: Front Endocrinol (Lausanne). 2026 Apr 22;17:1803726. doi: 10.3389/fendo.2026.1803726 (PMC13143533; doi:10.3389/fendo.2026.1803726)
Supplement: Supplementary file 1 [file DataSheet1.docx]

**Supplemental Table 1** Associations between the sperm DFI, ROS, and MMP and AZS severity

|  | Mild (PR ≥ 20%, n=115) | Moderate (10% ≤ PR < 20%, n=115) | Severe (PR < 10%, n=90) |
| --- | --- | --- | --- |
| DFI (%) | 23.6 (15.6, 32.3) | 25.8 (17.2, 42.6) ^d^ | 36.1 (20.6, 60.8) ^ac^ |
| ROS (%) | 1.8 (1.0, 2.8) | 2.1 (1.1, 3.6) ^d^ | 4.0 (2.3, 6.0) ^ab^ |
| MMP (%) | 57.8 (48.2, 64.9) | 52.7 (44.4, 65.1) ^d^ | 38.2 (28.3, 49.3) ^ab^ |

Note: Data are expressed as median (interquartile range). a, compared with the mild AZS group, *p* < 0.001; b, compared with the moderate AZS group, *p* < 0.001; c, compared with the moderate AZS group, *p* < 0.01; d, compared with the mild AZS group, *p* > 0.05

**Supplemental Table 2** Correlation analysis between sperm DFI, ROS, and MMP

|  | DFI | | ROS | | MMP | |
| --- | --- | --- | --- | --- | --- | --- |
|  | r | *p* | r | *p* | r | *p* |
| DFI | - | - | - | - | - | - |
| ROS | 0.255 | < 0.001 | - | - | - | - |
| MMP | -0.232 | < 0.001 | -0.531 | < 0.001 | - | - |

Note: *P* <0.05 represents significant differences.

**Supplemental Table 3** Comparison of baseline characteristics of women between high DFI group and low DFI group

|  | High DFI group  (≥ 25%) | Low DFI group  (< 25%) | *t* | *p* | High ROS group  (≥ 2.5%) | Low ROS group  (< 2.5%) | *t* | *p* | High MMP group  (≥ 50%) | Low MMP group  (< 50%) | *t* | *p* |
| --- | --- | --- | --- | --- | --- | --- | --- | --- | --- | --- | --- | --- |
| Cases | 64 | 79 |  |  | 55 | 88 |  |  | 86 | 57 |  |  |
| Duration of infertility (years) | 3.41±2.69 | 3.66±2.83 | 0.541 | 0.589 | 3.35±2.82 | 3.67±2.74 | 0.683 | 0.496 | 3.60±2.88 | 3.46±2.60 | -0.314 | 0.754 |
| Female age (years) | 31.83±2.50 | 31.71±2.77 | 0.267 | 0.790 | 31.76±2.72 | 31.76±2.61 | -0.005 | 0.996 | 31.63±2.64 | 31.96±2.66 | 0.745 | 0.458 |
| Female BMI (kg/m²) | 23.44±3.55 | 23.25±3.28 | 0.306 | 0.760 | 23.71±3.29 | 23.12±3.45 | -1.003 | 0.318 | 23.08±3.10 | 23.75±3.77 | 1.167 | 0.245 |
| Oocytes (number) | 13.44±6.80 | 12.04±5.57 | -1.353 | 0.178 | 11.89±4.40 | 13.15±7.03 | 1.315 | 0.191 | 12.48±5.76 | 12.95±6.78 | 0.445 | 0.657 |
| FSH (mIu/mL) | 6.63±2.89 | 7.31±3.46 | 1.258 | 0.210 | 6.86±2.80 | 7.10±3.48 | 0.422 | 0.674 | 7.16±3.03 | 6.78±3.52 | -0.686 | 0.494 |
| E2 (pg/mL) | 95.72±49.23 | 55.60±46.52 | -3.165 | 0.002 | 105.75±80.96 | 65.84±46.49 | -3.329 | 0.001 | 94.48±68.66 | 72.38±60.89 | 2.018 | 0.045 |
| LH (mIu/mL) | 7.77±4.11 | 7.63±4.41 | -0.113 | 0.910 | 6.82±4.17 | 9.09±8.34 | -1.554 | 0.125 | 6.61±3.96 | 9.33±10.24 | 1.916 | 0.060 |
| P (ng/mL) | 6.67±4.81 | 4.68±2.72 | -1.318 | 0.191 | 6.71±3.88 | 4.73±2.62 | -1.212 | 0.229 | 5.33±2.93 | 6.01±3.36 | 0.451 | 0.652 |
| AMH (ng/mL) | 3.22±2.60 | 2.99±2.37 | 0.538 | 0.591 | 2.87±2.19 | 3.23±2.63 | 0.860 | 0.391 | 3.06±2.46 | 3.14±2.51 | 0.184 | 0.854 |
| HCG daily endometrial thickness (mm) | 10.71±1.44 | 10.28±1.56 | -1.703 | 0.091 | 10.73±1.33 | 10.31±1.61 | -1.628 | 0.106 | 10.43±1.61 | 10.53±1.37 | 0.367 | 0.714 |

Note: Data are expressed as mean (standard deviation). FSH: Follicle-Stimulating Hormone; E2: estradiol; LH: Luteinizing hormone; AMH: Anti Mullerian hormone.

**Supplemental Table 4** Comparison of embryonic development and IVF pregnancy outcomes between high MMP\low DFI group and high DFI\low MMP group

|  | High MMP\low DFI  (MMP≥ 50% & DFI < 25%) | High DFI\low MMP  (MMP < 50% & DFI ≥ 25%) | χ2 | *p* |
| --- | --- | --- | --- | --- |
| cases | 54 | 32 |  |  |
| Fertilization rate (%) | 84.0  (508/605) | 74.2  (291/392) | 14.156 | < 0.001 |
| Cleavage rate (%) | 97.4  (495/508) | 97.6  (284/291) | 0.018 | 0.894 |
| High-quality embryo rate (%) | 37.0  (183/495) | 39.8  (113/284) | 0.609 | 0.435 |
| Blastocyst formation rate (%) | 54.8  (193/352) | 49.4  (116/235) | 1.69 | 0.194 |
| Pregnancy rate (%) | 70.4  (38/54) | 37.5  (12/32) | 8.920 | 0.003 |
| Miscarriage rate (%) | 7.9  (3/38) | 16.7  (2/12) | 0.780 | 0.582 |
| Live birth rate (%) | 64.8  (35/54) | 31.3  (10/32) | 21.258 | < 0.001 |

Note: Data are expressed as number (percent), *p* <0.05 represents significant differences.


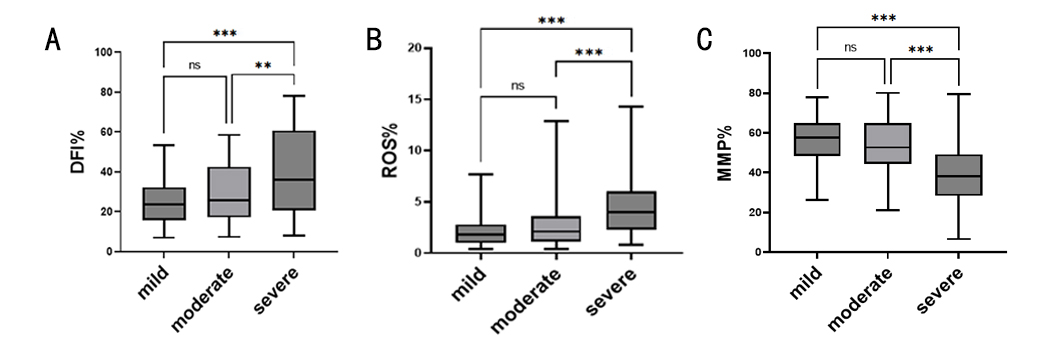


**Supplementary Figure.** Associations between the sperm DFI, ROS, and MMP and the severity of AZS

(A) Comparison the sperm DFI between groups. (B) Comparison the sperm ROS between groups. (C) Comparison the sperm MMP between groups. ns, *p* > 0.05; **, *p* < 0.01; ***, *p* < 0.001
